# Clavien–Dindo, comprehensive complication index and classification of intraoperative adverse events: a uniform and holistic approach in adverse event registration for (deep) endometriosis surgery

**DOI:** 10.1093/hropen/hoad019

**Published:** 2023-05-11

**Authors:** Jeroen Metzemaekers, Lotte Bouwman, Marit de Vos, Kim van Nieuwenhuizen, Andries R H Twijnstra, Maddy Smeets, Frank Willem Jansen, Mathijs Blikkendaal

**Affiliations:** Department of Gynecology/Endometriosis, Leiden University Medical Center, Leiden, The Netherlands; Department of Gynecology/Endometriosis, Leiden University Medical Center, Leiden, The Netherlands; Department of Gynecology/Endometriosis, Leiden University Medical Center, Leiden, The Netherlands; Department of Gynecology/Endometriosis, Leiden University Medical Center, Leiden, The Netherlands; Department of Gynecology/Endometriosis, Leiden University Medical Center, Leiden, The Netherlands; Department of Gynecology/Endometriosis, Leiden University Medical Center, Leiden, The Netherlands; Department of Gynecology/Endometriosis, Leiden University Medical Center, Leiden, The Netherlands; Department of Biomechanical Engineering, Delft University of Technology, Delft, The Netherlands; Department of Gynecology/Endometriosis, Leiden University Medical Center, Leiden, The Netherlands

**Keywords:** deep endometriosis, endometriosis, complications, adverse events, Clavien–Dindo

## Abstract

**STUDY QUESTION:**

What is the additional value of the comprehensive complication index (CCI) and ClassIntra system (classification for intraoperative adverse events (ioAEs)) in adverse event (AE) reporting in (deep) endometriosis (DE) surgery compared to only using the Clavien–Dindo (CD) system?

**SUMMARY ANSWER:**

The CCI and ClassIntra are useful additional tools alongside the CD system for a complete and uniform overview of the total AE burden in patients with extensive surgery (such as DE), and with this uniform data registration, it is possible to provide greater insight into the quality of care.

**WHAT IS KNOWN ALREADY:**

Uniform comparison of AEs reported in the literature is hampered by scattered registration. In endometriosis surgery, the usage of the CD complication system and the CCI is internationally recommended; however, the CCI is not routinely adapted in endometriosis care and research. Furthermore, a recommendation for ioAEs registration in endometriosis surgery is lacking, although this is vital information in surgical quality assessments.

**STUDY DESIGN, SIZE, DURATION:**

A prospective mono-center study was conducted with 870 surgical DE cases from a non-university DE expertise center between February 2019 and December 2021.

**PARTICIPANTS/MATERIALS, SETTING, METHODS:**

Endometriosis cases were collected with the EQUSUM system, a publicly available web-based application for registration of surgical procedures for endometriosis. Postoperative adverse events (poAEs) were classified with the CD complication system and CCI. Differences in reporting and classifying AEs between the CCI and the CD were assessed. ioAEs were assessed with the ClassIntra. The primary outcome measure was to assess the additional value toward the CD classification with the introduction of the CCI and ClassIntra. In addition, we report a benchmark for the CCI in DE surgery.

**MAIN RESULTS AND THE ROLE OF CHANCE:**

A total of 870 DE procedures were registered, of which 145 procedures with one or more poAEs, resulting in a poAE rate of 16.7% (145/870), of which in 36 cases (4.1%), the poAE was classified as severe (≥Grade 3b). The median CCI (interquartile range) of patients with poAEs was 20.9 (20.9–31.7) and 33.7 (33.7–39.7) in the group of patients with severe poAEs. In 20 patients (13.8%), the CCI was higher than the CD because of multiple poAEs. There were 11 ioAEs reported (11/870, 1.3%) in all procedures, mostly minor and directly repaired serosa injuries.

**LIMITATIONS, REASONS FOR CAUTION:**

This study was conducted at a single center; thus, trends in AE rates and type of AEs could differ from other centers. Furthermore, no conclusion could be drawn on ioAEs in relation to the postoperative course because the power of this database is not robust enough for that purpose.

**WIDER IMPLICATIONS OF THE FINDINGS:**

From our data, we would advise to use the Clavien–Dindo classification system together with the CCI and ClassIntra for a complete overview of AE registration. The CCI appeared to provide a more complete overview of the total burden of poAEs compared to only reporting the most severe poAEs (as with CD). If the use of the CD, CCI, and ClassIntra is widely adapted, uniform data comparison will be possible at (inter)national level, providing better insight into the quality of care. Our data could be used as a first benchmark for other DE centers to optimize information provision in the shared decision-making process.

**STUDY FUNDING/COMPETING INTEREST(S):**

No funding was received for this study. The authors have no conflicts of interest to declare.

**TRIAL REGISTRATION NUMBER:**

N/A.

WHAT DOES THIS MEAN FOR PATIENTS? Quality assessment and follow-up are important in any surgical procedure. This study, which focused on endometriosis, investigated whether using the comprehensive complication index (CCI) (reports complications on an easy scale from 0 to 100) and ClassIntra (reports intraoperative complications) classification systems, in addition to Clavien–Dindo (CD) (internationally leading system for postoperative complications), would give a better view of the total burden of complications (adverse events) that patients may experience during their medical treatments, compared to using CD alone. We found that using the CD system together with the CCI and the ClassIntra provides a complete overview of the adverse events that patients may experience during their medical treatments. This means that doctors and medical professionals can accurately identify, record, and categorize any complications that arise, ensuring that patients receive the best possible care. By using this system, doctors can identify any issues quickly, which can lead to faster treatment and better outcomes for patients. Overall, the use of the CD classification system, together with the CCI and ClassIntra, benefits patients by providing a clear and complete understanding of their medical treatments and any complications that may arise. Comparisons at a national and international level also then become possible, benefiting patients worldwide.

## Introduction

Quality assessment and follow-up are important in surgery. Quality assessment and registration of healthcare, including patient-reported outcome measures, could improve healthcare outcomes (Øvretveit *et al.*, 2017; Pop *et al.*, 2019). A vital part of this follow-up is the reporting of clinical outcomes and the registration of adverse events (AEs). Correct registration makes it possible to evaluate care at the individual patient level, but also facilitates data analysis at group level to gain insight and create the possibility to compare quality of care. Correct data analysis at group level can provide insight into acknowledging and recognizing deviating trends that can be reacted to in time, if necessary, to prevent future AEs.

Different systems can be used for the AE registration; however, the Clavien–Dindo (CD) system is currently the most used, validated, and accepted standard to classify perioperative AEs (Dindo *et al.*, 2004).

The CD system consists of five grades of severity, whereby Grade I is low and Grade V is death. A strength of the CD system is that it is widely accepted in surgery, making data comparison possible. Limitations of the CD are the fact that the data are ordinal, making data analysis difficult between two treatment options (a summative interpretation is not possible). Furthermore, because a weighting system is lacking, it is not possible to compare the impact of multiple minor AEs against one major AE. Additionally, for the ease of handling in AE registration (e.g. annual reports), it is common to capture only the AE with the highest grade (Eisenberg *et al.*, 2010; Kadlec *et al.*, 2013; Dumitra *et al.*, 2018). This automatically leads to data loss (incomplete overview of total AE burden) in patients with combinations of multiple AEs and, therefore, failure in presenting the actual morbidity of AEs.

To overcome these shortcomings, the comprehensive complication index (CCI) (Slankamenac *et al.*, 2013) was created in 2013. Based on the CD, the CCI can be used to calculate the impact of all cumulative AEs within a single patient using an algorithm. The overall burden of AE per patient is reflected in a single number on a scale from 0 (no AE) to 100 (death). This enables the comparison of patients with a single AE to patients with multiple AEs. Furthermore, the CCI provides a more accurate selection of those patients that need to be discussed in the morbidity and mortality meetings. Patients with CCI values of ≥33.7 are considered severe (Slankamenac *et al.*, 2013), and this cutoff point could be used for the selection of severe cases.

However, it should be noted that the CD and CCI systems are only for postoperative adverse events (poAEs). Intraoperative adverse events (ioAEs) that are solved during surgery cannot be classified by CD. ioAEs are also important to register and classify, as these events are associated with adverse outcomes in the postoperative course (Kaafarani *et al.*, 2014; Ramly *et al.*, 2015; Bohnen *et al.*, 2017). Furthermore, with the implementation of new surgical techniques (e.g. robotic surgery), the documentation of ioAEs will provide insight into the safety of the newly introduced techniques: this insight will not emerge with only the documentation of poAEs. Surprisingly, only recently (2020), the group of Dell-Kuster developed and validated a classification system named ClassIntra for reporting ioAEs (Table 1), which was developed after the CD system (Dell-Kuster *et al.*, 2020).

**Table 1.** ClassIntra version 1.0 for reporting intraoperative adverse events.

	Definition	Examples
Grade 0	No deviation from the ideal intraoperative course	
Grade I	Any deviation from the ideal intraoperative course Without the need for any additional treatment or intervention Patient asymptomatic or mild symptoms	Bleeding: bleeding above average from small-caliber vessel: self-limiting or definitively manageable without additional treatment than routine coagulation Injury: minimal serosal intestinal lesion, not requiring any additional treatment Cautery: small burn of the skin, no treatment necessary Arrhythmia: arrhythmia (e.g. extrasystoles) without relevance
Grade II	Any deviation from the ideal intraoperative course With the need for any additional minor treatment or intervention Patient with moderate symptoms, not life-threatening and not leading to permanent disability	Bleeding: bleeding from medium caliber artery or vein, ligation; use of tranexamic acid Injury: non-transmural intestinal lesion requiring suture(s) Cautery: moderate burn requiring non-invasive wound care Arrhythmia: arrhythmia requiring administration of antiarrhythmic drug, no hemodynamic effect
Grade III	Any deviation from the ideal intraoperative course With the need for any additional moderate treatment or intervention Patient with severe symptoms, potentially life-threatening and/or potentially leading to permanent disability	Bleeding: bleeding from large caliber artery or vein with transient hemodynamic instability, ligation, or suture; blood transfusion Injury: transmural intestinal lesion requiring segmental resection Cautery: severe burn requiring surgical debridement Arrhythmia: arrhythmia requiring administration of antiarrhythmic drug, transient hemodynamic effect
Grade IV	Any deviation from the ideal intraoperative course With the need for any additional major and urgent treatment or intervention Patient with life-threatening symptoms and/or leading to permanent disability	Bleeding: life-threatening bleeding with splenectomy; massive blood transfusion; ICU stay Injury: injury of central artery or vein requiring extended intestinal resection Cautery: life-threatening burn injury by cautery leading to fire requiring ICU treatment Arrhythmia: arrhythmia requiring electroconversion, defibrillation, or admission to the ICU
Grade V	Any deviation from the ideal intraoperative course With intraoperative death of the patient	

For the classification of poAEs in endometriosis surgery, the Clavien–Dindo and use of the CCI have been recommended since 2016 (Vanhie *et al.*, 2016); however, there are currently no publications on the use of the CCI. Also, no data could be found on the topic on how to report ioAEs in surgery for deep endometriosis (DE), something that is vital for a complete overview of the surgical quality assessment, especially because DE is particularly associated with high AE rates, owing to the complexity of the disease and surgery. However, it is challenging to gain proper insight into how severe some AEs are, especially in patients with multiple AEs.

This study aims to assess the hypothesis that additional use of the CCI and ClassIntra classification systems will provide a better view of the total burden of AEs compared to using CD alone. The objective is to establish a standardized registration of AEs in (deep) endometriosis surgery, facilitating comparisons at both national and international levels.

## Materials and methods

This study used prospectively collected data on surgical cases with endometriosis or DE from a Dutch DE expertise center (Endometriose in Balans, Haaglanden Medical Centre, the Hague, the Netherlands). Data inclusion took place from February 2019 through December 2021 by the EQUSUM application (Metzemaekers *et al.*, 2020).

For this study, we used the definition of DE described by the international working group of American Association of Gynecologic Laparoscopists, European Society for Gynaecological Endoscopy, ESHRE, and World Endometriosis Society in 2021 (Tomassetti *et al.*, 2021), which is classified with the Enzian-based system on clinical expertise combined with radiological and intraoperative findings. Patients who underwent a diagnostic laparoscopy were excluded from the study. For adenomyosis, we used the Morphological Uterus Sonographic Assessment (MUSA) for classification (Van den Bosch *et al.*, 2019).

### Case characteristics

Registered data included: general data on patients’ characteristics, surgical indication, surgical procedure, previous abdominal surgery, and accurate localization of DE lesions and adhesion scoring. The EQUSUM (Metzemaekers *et al.*, 2020) application automatically generated the following classifications: revised American Society for Reproductive Medicine (rASRM) (American Society for Reproductive Medicine, 1997), Enzian (Keckstein *et al.*, 2003), and Endometriosis Fertility Index (EFI) scores (Adamson and Pasta, 2010). The Enzian scores were assigned according to the original manuscript (Tuttlies *et al.*, 2005).

### Adverse event registration

AEs were documented until 42 days post-procedure according to the CD classification (Dindo *et al.*, 2004). Surgical poAEs were defined as any event that represents a deviation in the expected postoperative course (Dindo and Clavien, 2008). The validated CCI (Slankamenac *et al.*, 2013) was calculated with the following: CCI formula CCI^®^ = √ (wC1 + wC2 + wC*x*)/2 where wC stands for weight of complication. CD Grade I = wC 300, Grade II = wC 1750, Grade IIIa = wC 2750, Grade IIIb = wC 4550, Grade IVa = wC 7200, and Grade IVb = wC 8550. CD Grade V always results in CCI^®^ 100.

ioAEs were classified by the validated ClassIntra reporting system and defined as ‘any deviation from the ideal intraoperative course between skin incision and skin closure, and includes events related to surgery and anaesthesia’ (Dell-Kuster *et al.*, 2020). Table 1 shows the classification of ioAEs, ranging from Grades I to V.

### Statistical analysis

The Statistical Package for Social Sciences (SPSS, Chicago, IL, USA), version 28.0 was used for analysis. Categorical variables were described as frequencies with percentages. For normally distributed or skewed data, data were presented as mean with SD or median with interquartile range (IQR), respectively. The Shapiro–Wilk test was used to determine the distribution of the data. For group comparisons with parametric data, an independent Student’s T-test was performed, while for non-parametric data, the Mann–Whitney test was performed. A two-tailed *P*-value of <0.05 was considered as statistically significant.

### Ethical approval

Ethical approval was given by the Medical Ethics Committee of the Leiden University Medical Centre for the use of the anonymous data in the EQUSUM database (LUMC) (G20.019).

## Results

A total of 870 cases met the inclusion criteria (85.9% DE cases, 14.1% endometriosis cases). The mean age of the women was 36.4 (SD 7.1) years, with a mean BMI of 24.9 (SD 4.5) kg/m^2^ (Table 2). Pain was the primary indication for surgery in 87.1% of the cases. The median (IQR) operating time was 75.0 (40–120) min, with a median (IQR) blood loss of 50 ml (0–50) (range 0–1700 ml).

**Table 2.** Baseline characteristics of patients with surgical (deep) endometriosis.

Baseline characteristics	Total group	Cases without poAEs	Cases with poAEs	*P*-value
	n = 870	n = 725 (83.3%)	n = 145 (16.7%)	
Age, mean (SD)	36.42 (7.1)	34.10 (7.1)	36.03 (7.2)	<0.01
BMI in kg/m^2^, mean (SD)	24.89 (4.5)	24.79 (4.4)	25.39 (4.7)	0.16
Type of endometriosis, (n, %)				
Non-DE endometriosis	123 (14.1)			
Deep endometriosis	747 (85.9)			
Previous procedures (%)				0.09
0 procedures	376 (43.2)	320 (44.1)	56 (38.6)	
1 procedure	275 (31.6)	228 (31.4)	47 (32.4)	
2 procedures	109 (12.5)	94 (13.0)	15 (10.3)	
>2 procedures	110 (12.6)	83 (11.4)	27 (18.6)	
Indication surgery (%)				0.09
Pain	758 (87.1)	625 (86.2)	133 (91.7)	
Fertility	95 (10.9)	86 (11.9)	9 (6.2)	
Cyst formation	7 (0.8)	7 (1.0)	0 (–)	
Organ damage	5 (0.6)	3 (0.4)	2 (1.4)	
Abnormal uterine bleeding	3 (0.3)	3 (0.4)	0 (0)	
Other	2 (0.2)	1 (1.3)	1 (0.7)	
Type of procedure, (n, %)				N/A
Laparoscopy	867 (99.7)	724 (99.9)	143 (98.6)	
Laparotomy	3 (0.3)	1 (0.1)	2 (1.4)	
Hysterectomy				
Yes	253 (29.1)	196 (27.0)	57 (39.3)	<0.01
No	617 (70.9)	529 (73.0)	88(60.7)	
Operating time in minutes, median (IQR)	75.0 (40–120)	70 (40–120)	90 (45–150)	<0.01
Blood loss in ml, median (IQR)	50 (0–50)	50 (0–50)	50 (10–50)	<0.01
Adhesion score median (IQR)	4 (1–9)	4.0 (1–8)	5.5 (2–10)	0.02
Pouch of Douglas obliteration				
Partial obliteration	99 (35.0)	88 (12.1)	11 (7.6)	0.02
Total obliteration	184 (65.0)	142 (19.6)	42 (29.0)	
**rASRM stage %** ^a^	I: 22.2%			
	II: 16.8%			
	III: 19.9%			
	IV: 29.1%			
**EFI score %** ^b^	0–3: 1.7%			
	4: 3.2%			
	5: 2.5%			
	6: 3.8%			
	7–8: 14.6%			
	9–10: 12.5%			
**Enzian classification**	**Total, N (%)**	**<1 cm**	**1-3 cm**	**>3 cm**
		**N (%)**	**N (%)**	**N (%)**
Compartment A	160 (18.4)	12 (7.5)	57 (35.6)	91 (56.9)
Compartment B left	417 (47.9)	62 (14.9)	229 (54.9)	126 (30.2)
Compartment B right	379 (43.6)	62 (16.4)	227 (59.9)	90 (23.7)
Compartment C	263 (30.2)	21 (8.0)	93 (35.4)	149 (56.7)
Compartment C (high)^c^	101 (11.6)	6 (5.9)	50 (49.5)	45 (44.6)
Compartment FA	499 (57.4)			
Focal	186 (21.4)			
Diffuse	290 (33.3)			
Adenomyosis	18 (2.1)			
Other	5 (0.6)			
Compartment FB	113 (13.0)	29 (25.7)	53 (46.9)	31 (27.4)
Compartment FU left^d^	101 (11.6)	15 (14.9)	56 (55.4)	30 (29.7)
Compartment FU right^d^	74 (8.5)	12 (16.2)	45 (60.8)	17 (23.0)
Compartment FI	165 (19)			
Compartment FO	47 (5.4)			
Peritoneal involvement	618 (71.0)	83 (13.4)	182 (29.4)	353 (57.1)
Hydronephrosis				
Left	11 (1.3)			
Right	10 (1.1)			
Appendix involvement	76 (8.7)	16 (21.1)	51 (67.1)	9 (11.8)
**Surgical procedures**		**Shaving, n (%)**	**Discoid, n (%)**	**Segment, n (%)**
Urethral catheter use	417 (47.9)			
Rectum surgery (Enzian C)	223 (25.6)^e^	72 (32.3)	21 (9.4)	129 (57.8)
Rectum surgery high	85 (9.8)^e^	14 (16.5)	9 (10.6)	61 (71.8)
Ileocecal surgery	12 (1.4)	2 (16.7)	1 (8.3)	9 (75.0)
Bladder surgery	102 (11.7)			
Partial full thickness	20 (19.6)			
Resection full thickness	29 (28.4)			
Shave	53 (52)			
Ureter surgery				
Left	101 (11.6)			
Right	69 (7.9)			
Tuba surgery				
Left	52 (6.0)			
Right	22 (2.5)			
Ovary surgery				
Left	249 (28.6)			
Right	210 (24.1)			

a 12.1% of the cases (n = 105) did not have a rASRM stage, while there was endometriosis outside the internal reproductive organs and peritoneum, not possible to classify with the rASRM.

b 61.6% of the cases (n = 536) did not have an EFI score, while the indication for surgery was not fertility.

c Cranial to sigmoid junction.

d FU involvement is a combination of intrinsic and extrinsic endometriosis involvement.

e Surgical technique in one case not further specified.

IQR: interquartile range; rASRM: revised American Society for Reproductive Medicine; EFI: Endometriosis Fertility Index; poAE: postoperative adverse event; Compartment A: Enzian vagina; Compartment B: Enzian ligaments; Compartment C: Enzian rectum; FB: Enzian bladder; FA: Enzian adenomyosis; FU: Enian ureter; FI: Enzian intestine; FO: Enzian other.

Independent Student’s T-test and the Mann–Whitney test were used for statistical analysis.

### Postoperative adverse events

Table 3 shows the total number of poAEs reported (n = 168 in 145 cases), among which Grade II was most often present (10.3%). A total of 125 (86.2%) patients had a single poAE, 17 (11.7%) patients had 2 poAEs, and there were 3 (2.1%) patients with 3 poAEs. Overall, a total of 870 procedures were registered, of which 145 procedures resulted in one or more poAEs, resulting in a poAE rate of 16.7% (145/870), of which 36 (4.1%) cases were classified as severe.

**Table 3.** Postoperative adverse events in women following surgery for (deep) endometriosis.

	N (%)	% within poAE
Total number of poAEs,^a^ n (%)	168 (19.3)	100
Grade I	34 (3.9)	20.2
Grade II	90 (10.3)	53.6
Grade IIIa	5 (0.5)	3.0
Grade IIIb	36 (4.1)	21.4
Grade IVa	2 (0.2)	1.2
Grade IVb	1 (0.1)	0.6
Grade V	0	0.0
Required re-operation	36 (4.1)	21.4
Number of poAEs within patients, n (%)	145 (16.7)	100
1 poAE	125 (14.4)	74.4
2 poAEs	17 (2.0)	11.7
3 poAEs	3 (0.3)	2.1
Most severe poAE by CD, n (%)	145 (16.7)	100
Grade I	30 (3.4)	20.7
Grade II	77 (8.9)	53.1
Grade IIIa	2 (0.2)	1.4
Grade IIIb	33 (3.8)	22.8
Grade IVa	2 (0.2)	1.4
Grade IVb	1 (0.1)	0.7
‘missed’ AEs by only reporting the most severe AEs	23 (2.6)	13.7
Disability reported	14 (1.6)	9.7
Overall AEs rate, n (%)	145/870 (16.7)	100
Severe poAE^b^ in total population, n (%)	36/870 (4.1)	24.8
Median CCI (IQR)	20.9 (20.9–31.7)	—
CCI ≥ 33.7 (IQR)	33.7 (33.7–39.7)	—
≥1 poAEs (per year)		
2019, n = 218 (mean CCI 25.4, median 20.9)	58 (21.0)	
2020 n = 269 (mean CCI 22.2, median 20.9)	40 (14.9)	
2021 n = 325 (mean CCI 20.6, median 20.9)	47 (14.5)	

a Some patients have more than one postoperative AE.

b CCI ≥ 33.7.

poAE: postoperative adverse event; CCI: comprehensive complication index; CD: Clavien–Dindo.

n = 168 single adverse events in 145 cases.

Table 4 presents the type of poAE in relation to the severity. The majority of poAEs consisted of infections at organ level (35.1%), of which the majority involved a cystitis (29.2%). Of the infection at organ level, most of the severity was scored as a Grade II severity. Local infections were reported in 16.7% of cases, mostly surgical site infections (13.3%). Anastomosis leakage occurred in 5.4% of the poAEs (1.0% in total population), and all of these poAEs were scored with severity IIIB. The highest poAE was a Grade IVB and was a bowel injury. Acute kidney failure was reported in 3.6% (n = 6).

**Table 4.** Type of postoperative adverse events and severity score by the Clavien–Dindo classification.

AEs, descriptions	n	% in cases with poAE	% in total population	I	II	IIIa	IIIb	IVa	IVb	V
				n (%)	n (%)	n (%)	n (%)	n (%)	n (%)	n (%)
Local infection	28	16.7	3.2	7 (25.0)	21 (75.0)					
Surgical site	22	13.1	2.5							
Other	6	3.6	0.7							
Infection organ level	59	35.1	6.8	1 (1.7)	55 (93.2)	2 (3.4)		1 (1.7)		
Cystitis	49	29.2	5.6							
Pyelonephritis	4	2.4	0.5							
Pneumonia	4	2.4	0.5							
Other	2	1.2	0.2							
Systemic infection	1	0.6	0.1				1 (100)			
Peritonitis	1									
Bowel injury	5	3.0	0.6				4 (80.0)		1 (20.0)	
Perforation	5									
Bladder injury	3	1.8	0.3	1 (33.3)			2 (66.6)			
Ureter injury	4	2.4	0.5	1 (25.0)		1 (25.0)	2 (50.0)			
Fistula injury	6	3.6	0.7				6 (100.0)			
Recto-vaginal	5	2.8	0.6							
Other	1	0.6	0.1							
Wound dehiscence	5	3.0	0.6				5 (100.0)			
Incision site	1	0.6	0.1							
Vaginal cuff dehiscence	1	0.6	0.1							
Other	3	1.8	0.3							
Anastomotic leakage	9	5.4	1.0				9 (100.0)			
Hemorrhage/hematoma	10	6.0	1.1	5 (50.0)	2 (20.0)		3 (30.0)			
Thrombosis/embolism	7	4.2	0.8	4 (57.1)	3 (42.9)					
Pulmonary embolism	1	0.6	0.1							
Thrombophlebitis	6	3.6	0.7							
Urinary retention	6	3.6	0.7	5 (83.3)	1 (16.7)					
Ileus	2	1.2	0.2		2 (100.0)					
Kidney dysfunction	7	4.2	0.8	4 (57.1)	1 (14.3)	1 (14.3)	1 (14.3)			
Acute kidney failure	6	3.6	0.7							
Other	1	0.6	0.1							
Disfunction, ureter	1	0.6	0.1		1 (100.0)					
Other AEs	15	8.9	1.7	6 (40.0)	4 (26.7)	1 (6.7)	3 (20.0)	1 (6.7)		
Total number of poAEs, n (%)	168 (100)			34 (20.2)	90 (53.6)	5 (3.0)	36 (21.4)	2 (1.2)	1 (0.6)	0 (0)

poAE: postoperative adverse event.

### The CCI

**Figure 1.**
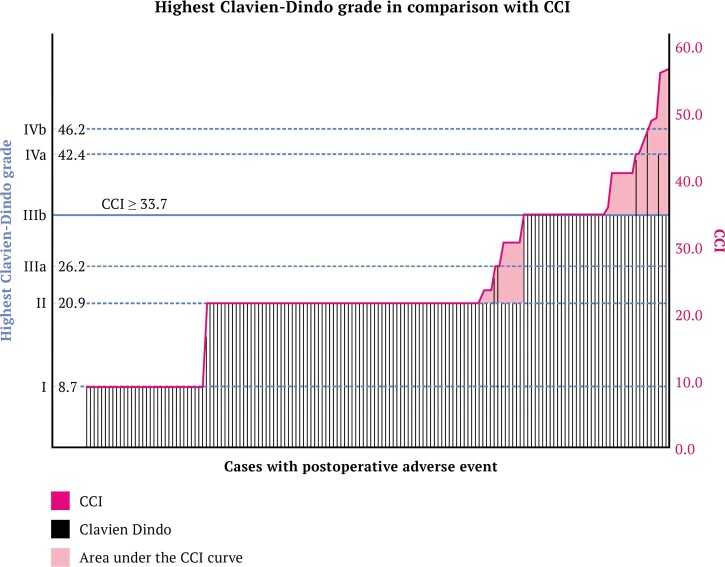
**All patients with endometriosis with postoperative adverse events in relation to the most severe Clavien–Dindo classification and comprehensive complication index.** On the left axis, the severity of the Clavien–Dindo (CD) is shown, while on the right axis, the comprehensive complication index (CCI) is shown. The pink line presents the CCI and the black vertical lines present the highest CD grade. N = 145. The area under the CCI curve (pink) shows the increase in CCI of patients with multiple postoperative adverse events (poAEs) compared to only reporting the most severe AE (as would apply if only the CD system was used). A CCI ≥33.7 is considered as a severe poAE. To illustrate, the case with the most severe poAE has a CCI of 54.4. The highest CD is a Grade IIIb poAE, which would correspond to a CCI of 33.7. The gap between 33.7 and 54.4 is explained by multiple other poAEs that are not captured if only the CD system is used.

Table 3 shows the median CCI of 20.9 (IQR 20.9–31.7) for all poAEs. Looking at the patients with a severe poAE, a median CCI of 33.7 was reported (IQR 33.7–39.7). Figure 1 shows the patients with the highest reported CD poAE (black bars) in relation to the CCI (pink line). There are 36 cases (24.8%) with a CCI ≥33.7, and these are considered as severe poAEs (see reference line in Fig. 1). The area under the CCI curve (pink) shows the increase in CCI of patients with multiple poAEs compared to only reporting the most severe AE (as would apply if only the CD system was used). To illustrate, the case with the most severe poAE has a CCI of 54.4. The highest CD is a Grade IIIb poAE, which would correspond to a CCI of 33.7. The gap between 33.7 and 54.4 is explained by multiple other poAEs that are not captured if only the CD system is used. In 20 patients (13.8%), the CCI was higher than the CD because of multiple poAEs. As shown in Table 5, cases with multiple AEs increase in CCI compared to only reporting the highest CD. Case #1 increases in the CCI from 33.7 to 44.9, Case #2 increases from 33.7 to 47.6, and Case #3 remains the same while it is only a single AE.

**Table 5.** The three cases with major surgery and the corresponding CCI.

Adverse event, description, surgical approach	AE1	AE2	AE3	Highest CD	CCI total
	(CCI)	(CCI)	(CCI)	(CCI)	
#1 Endometriosis classification: Enzian A2B3C3, FA, FI Surgical procedure: hysterectomy + BSO, low rectal resection, discoid resection sigmoid	Surgical side infection II (20.9)	Cystitis II (20.9)	Anastomotic leakage IIIb (33.7)	IIIb (33.7)	44.9
#2 Endometriosis classification: Enzian A0B0C3, FA, FB, FI, FU	Cystitis II (20.9)	Ureter injury IIIa (26.2)	Anastomotic leakage IIIb (33.7)	IIIb (33.7)	47.6
Surgical procedure: ultra-low rectal resection, cecal resection, bladder surgery full thickness					
#3 Endometriosis classification: Enzian A3B2C3, FA, FI, FU	Anastomotic leakage IIIb (33.7)			IIIb (33.7)	33.7
Surgical procedure: hysterectomy + BSO, ultra-low rectal resection, ureterolysis, cecal discoid					

Compartment A: Enzian vagina; Compartment B: Enzian ligaments; Compartment C: Enzian rectum; FB: Enzian bladder; FA: Enzian adenomyosis; FU: Enian ureter; FI: Enzian intestine; BSO: bilateral salpingo-oophorectomy; CCI: comprehensive complication index.

The Enzian classification, surgical procedure, type of postoperative adverse event, most severe Clavien–Dindo (CD) score, and total CCI are shown.

### Intraoperative adverse events

In total, 11 (11/870, 1.3%) ioAEs arose and were corrected directly during surgery (Table 6). These included serosa injury (n = 6 of 870 (0.7%)), blood vessel injury (n = 2), uterus perforation (n = 1), rectal perforation (n = 1), and perforation of the sigmoid (n = 1). Of these, 10 (90.9%) ioAEs were staged as Grade II and 1 (9.1%) ioAE as Grade III because of 1700 ml blood loss. The most frequent ioAE was a directly sutured intestinal serosa lesion in 6 (54.5%) cases, followed by 2 (18.2%) cases with a bleeding. When summing up poAEs and ioAEs, this results in a total AE rate of 17.6% (n = 153). Eight patients (0.9%) only had an ioAE, 142 (16.3%) patients only had a poAE, and 3 (0.3%) patients had both an ioAE and a poAE. Of the three patients with both an ioAE and poAE, one patient showed a relation between the ioAE and the poAE; this was serosa injury of the rectum that was sutured during the surgery, and this patient developed a peritonitis based on a perforation that was furthermore complicated with respiratory distress.

**Table 6.** Intraoperative adverse events, classified by ClassIntra.

Intraoperative adverse events	n (%)	Grade by Classintra
Overall ioAE rate in population	11 (1.3)	
Intestinal serosa injury (directly repaired)	6 (54.5)	Grade II
Bleeding	2 (18.2)	Grade II (n = 1)
		Grade III (n = 1)
Perforation of uterus, no macroscopic lesion	1 (9.1)	Grade II
Rectal perforation	1 (9.1)	Grade II
Perforation of sigmoid	1 (9.1)	Grade II

**Total number of patients with poAEs and/or ioAEs, n (%)^a^**	153 (17.6)	

Only intraoperative, n (%)	8 (0.9)	
Only postoperative, n (%)	142 (16.3)	
Both intra- and postoperative, n (%)	3 (0.3)	

a Because of rounding there is a difference of 0.1% between total percentage and subgroups.

ioAE: intraoperative adverse event; poAE: postoperative adverse event.

N = 870 surgeries.

## Discussion

This study was performed to advocate uniform registration in the field of AEs in endometriosis surgery, and especially in DE, making use of the CD and CCI as well as the ClassIntra systems. This is one of the largest and most detailed studies on this topic, which could act as a first step toward benchmarking AEs in DE surgery.

### CCI in comparison with only using the CD

A high percentage of poAEs were registered, namely 16.7%. Easy discrimination between severe AEs is possible with the CCI (CCI ≥ 33.7). We found only 4.1% of patients with severe AEs, which are interesting to discuss in, for example, morbidity and mortality meetings. Furthermore, with the CCI, it is possible to calculate the total burden of AEs, instead of only reporting the most severe AE (as with CD). If we only used the most severe AE (which is common with the CD), 23 poAEs would not have been used in the total AE calculation (23/168, 13.7%). This is a significant part of the total AE burden.

As far as we know, there are no endometriosis studies where the CCI is used for the registration of poAEs. In our study, the median CCI was 20.9 (IQR 20.9–31.7) in the total cohort, and 33.7 (IQR 33.7–39.7) in the patients experiencing severe AEs. This is comparable with non-endometriosis literature: Tamini *et al.* (2021) established a median CCI of 20.9 (IQR 20.9–36.2) in a cohort who underwent surgery for colon cancer. Furthermore, in a recent publication by Haas *et al.* (2021) concerning bladder surgery, the median 30-day CCI was 22.6 (IQR 8.8–39.7).

The CCI also makes it easy to compare poAE rates over the years, something that is more challenging with an ordinal scale (CD). It was easy to show that the median CCI over the years did not significantly change. In contrast, with the CD alone, it was only possible to detect frequencies instead of comparing the total AE burden.

### Classifying intraoperative adverse events: ClassIntra

In our cohort, 11 ioAEs occurred, which were treated intraoperatively. These 11 ioAEs would not have been classified if only the CD system was used. For a complete overview in the quality assessment of surgical audits, these 11 ioAEs are also a significant part of the AEs that need to be classified. This is especially important when new surgical techniques are introduced. The ClassIntra could be a good tool in the debriefing period at the end of a surgical procedure. In this way, the registration is directly performed and the whole team could instantly reflect on any ioAE that occurred. Furthermore, the team can discuss strategies aimed at preventing any poAE that may occur as a result of the ioAE, such as increased monitoring for peritonitis in cases with intraoperative intestinal injuries. To highlight the literature gap on ioAE reporting, a critical appraisal of 46 randomized controlled surgical trials on AEs concluded that only 41% of the studies reported ioAEs, with 13% providing a definition and only 9% using a classification for these ioAEs (Rosenthal *et al.*, 2015). This shows that consensus is lacking, while there is a need for a widely applicable classification system. From our data, three cases showed both an ioAE and a poAE, and one case had a relation to both AEs (serosa injury and peritonitis). Others have illustrated that these types of ioAE are associated with adverse outcomes in the postoperative course (Bohnen *et al.*, 2017). This also shows how one event could trigger a cascade of AEs, either directly or more indirectly owing to increased patient vulnerability and case complexity (de Vos *et al.*, 2019).

A limitation of the ClassIntra system is, for example, the fact that the system relies heavily on the individual surgeon to identify and report complications, which can lead to under-reporting. However, the question about the subject ‘what is an adverse event’ does not only apply to ioAEs but also to poAEs. This question can be answered with Delphi methods, but that is beyond the scope of this research, though of great importance in the standard reporting of AEs.

This is the first AE study in endometriosis care that uses a total burden of all AEs (intra, post, single, and multiple AEs), which is a strength of our study. With this study, we hope to encourage other centers to also adopt these classification systems. Not only will this provide a more accurate and complete overview of AEs but also it will enable data comparison on multiple levels. A limitation of this study is the fact that it was performed in a single center, which limits the number of cases. However, even with a smaller number of cases, this study is able to illustrate how the addition of the CCI and ClassIntra systems would lead to a more comprehensive image of the complications that may arise in surgical endometriosis cases.

### Recommendations for clinical practice

In AE reporting, we recommend to use the Clavien–Dindo for poAEs, which aligns with international recommendations (Vanhie *et al.*, 2016). The usage of the CCI is also recommended by these same guidelines (Vanhie *et al.*, 2016); however, to our knowledge, no publications are available on this topic in relation to DE surgery. Therefore, we would like to promote the use of the CCI alongside the CD.

Owing to several instrument-specific advantages, the CCI appears to be a useful addition to the CD system because:

- it presents the total burden of poAEs per patient (cumulative formula);
- it uses a linear scale instead of an ordinal scale, which is beneficial for research/statistical purposes;
- there is easy discrimination of severe poAEs when using the cutoff point of CCI ≥33.7;
- we can compare patients with one poAE to patients with multiple poAEs; and
- it creates easy and interpretable data for benchmarking.

We acknowledge that implementing our recommendations will necessitate additional registration time for doctors, who already devote a significant amount of time to computer-based tasks rather than direct patient care. However, if the IT is properly designed and the registration is performed accurately, it will eventually save time and energy. For example, with the EQUSUM application, it is easy to perform statistics, while the syntax for all the calculations is already there. The CCI will automatically be generated after running the syntax and all other calculations for AE percentages. Proper use of the current and future technologies does have the potential to decrease workload instead of increasing it, even with more registration. Effort should be put into making it as easy as possible for doctors, and ‘reward’ them with easy and automatically generated monthly and annual reports for the morbidity and mortality meetings.

## Conclusion

This study showcases the advantages of integrating two supplementary systems—the CCI and Classintra system—in the reporting of AEs that occur during endometriosis surgery. Although the Clavien–Dindo classification is a commonly used tool for evaluating postoperative complications, it has limitations in capturing the entirety of AEs, particularly those that arise during surgery. In order to address this limitation, the CCI and Classintra system were developed, providing standardized methods for categorizing and reporting multiple and ioAEs. By utilizing these systems, a more inclusive and thorough summary of all AEs can be generated, leading to a more comprehensive understanding of patient outcomes and the potential to lessen the overall burden of AEs associated with surgery.

## Data Availability

The data underlying this article will be shared upon reasonable request to the corresponding author.
